# Towards phase-sensitive optical coherence tomography in smart laser osteotomy: temperature feedback

**DOI:** 10.1007/s10103-023-03886-z

**Published:** 2023-09-26

**Authors:** Arsham Hamidi, Yakub A. Bayhaqi, Ferda Canbaz, Alexander A. Navarini, Philippe C. Cattin, Azhar Zam

**Affiliations:** 1 Biomedical Laser and Optics Group (BLOG), Department of Biomedical Engineering, University of Basel, CH-4123 Allschwil, Switzerland; 2 Digital Dermatology, Department of Biomedical Engineering, University of Basel, CH-4123 Allschwil, Switzerland; 3 Center for Medical Image Analysis and Navigation (CIAN), Department of Biomedical Engineering, University of Basel, CH-4123, Allschwil, Switzerland; 4 Division of Engineering, New York University Abu Dhabi, Abu Dhabi, 129188 UAE; 5 Tandon School of Engineering, New York University, Brooklyn, NY 11201 USA

**Keywords:** Optical coherence tomography, Phase-sensitive OCT, Laser ablation, Laser osteotomy

## Abstract

Thermal effects during bone surgery pose a common challenge, whether using mechanical tools or lasers. An irrigation system is a standard solution to cool the tissue and reduce collateral thermal damage. In bone surgery using Er:YAG laser, insufficient irrigation raises the risk of thermal damage, while excessive water lowers ablation efficiency. This study investigated the potential of optical coherence tomography to provide feedback by relating the temperature rise with the photo-thermal expansion of the tissue. A phase-sensitive optical coherence tomography system (central wavelength of *λ*=1.288 μm, a bandwidth of 60.9 nm and a sweep rate of 104.17 kHz) was integrated with an Er:YAG laser using a custom-made dichromatic mirror. Phase calibration was performed by monitoring the temperature changes (thermal camera) and corresponding cumulative phase changes using the phase-sensitive optical coherence tomography system during laser ablation. In this experiment, we used an Er:YAG laser with 230 mJ per pulse at 10 Hz for ablation. Calibration coefficients were determined by fitting the temperature values to phase later and used to predict the temperature rise for subsequent laser ablations. Following the phase calibration step, we used the acquired values to predict the temperature rise of three different laser-induced cuts with the same parameters of the ablative laser. The average root-mean-square error for the three experiments was measured to be around 4 °C. In addition to single-point prediction, we evaluated this method’s performance to predict the tissue’s two-dimensional temperature rise during laser osteotomy. The findings suggest that the proposed principle could be used in the future to provide temperature feedback for minimally invasive laser osteotomy.

## Introduction

Osteotomy (bone surgery) is a surgical intervention involving machining processes such as drilling, sawing, and grinding the bone [1]. The mechanical stress that conventional tools apply to the surface of the bone is associated with several disadvantages, including poor surface evenness, limited cutting geometry, and extensive heat deposition [2]. Laser osteotomy, the proclaimed successor of mechanical saws and chisels, has the potential to revolutionize osteotomy. The unique advantages of using lasers, such as non-contact intervention, faster healing time, and the possibility of performing unrestricted cutting geometries, have garnered significant interest in applying this technology in medical settings [3–8]. Cortical bone is composed of approximately 13% water, 60% mineral in the form of hydroxyapatite crystallites, and 27% of organic matrix [9]. The absorption peak of different constituents of bone tissue in the mid-infrared wavelength range makes these lasers interesting for the ablation of bone tissue. For instance, CO_2_ laser at 10.6 μm with a high absorption peak in mineral composition and Er:YAG laser at 2.94 μm with a high absorption peak in water’s spectrum [10]. The ablation process with Er:YAG lasers assisted by water improves the ablation efficiency and surface morphology [11]. Several studies have shown the advantages of using Er:YAG lasers generating microsecond pulses to achieve deep bone ablation with minimum collateral thermal damage to the surrounding tissue. Laser ablation using an Er:YAG laser is known as photothermal ablation, where following the high absorption of the Er:YAG laser pulse in bone tissue, transferred energy to water molecule builds high pressure (~several hundred bars) which leads to a series of micro explosions by vaporization and consequently the removal of bone tissue [12–14]. Although this laser-tissue interaction can minimize collateral thermal damage and achieve a deeper cut, it also quickly dries out the surrounding tissue and can cause thermal damage [15]. Generally, photothermal damage can be divided into two main categories: reversible effects, such as hyperthermia, and irreversible damages, which encompass coagulation, vaporization, carbonization, and melting. The denaturation of proteins (coagulation) arises when the temperature within biological tissue reaches ≅60 °C. Localized temperature increases beyond 100 °C result in tissue vaporization, and when this vaporization process exhausts the tissue’s water content, it transforms the organic compounds of the tissue into carbon (carbonization) [16]. Melting occurs at temperatures surpassing 300°C. Consequently, irrigation is required during laser osteotomy to rehydrate and prevent thermal damage to the surrounding critical tissues [17]. However, because of the high absorption of Er:YAG laser in water, an excessive amount of water will work as a protective layer by absorbing the energy of the laser pulses, thereby reducing ablation efficiency [18]. To overcome this challenge, a non-contact feedback system is required to allow for pulsed irrigation instead of continuous irrigation during laser osteotomy.

To date, many methods have demonstrated potential for measuring temperature changes during laser treatments in cases where ablation (tissue removal) is not involved. These methods include magnetic resonance thermal imaging (MRTI), fiber optic sensors (FOS), and ultrasound (US) among others [17, 19–21]. Among these methods, the optoacoustic (OA) technique has shown promising results for achieving non-contact temperature measurements [22]. OA uses short laser pulses to generate pressure waves in the tissue. The Grüneisen parameter, a dimensionless parameter, is proportional to an increase in the pressure of the produced acoustic wave [23]. OA leverages the linear relation between the temperature and the tissue’s Grüneisen parameter for temperature measurement. This accuracy could be enhanced through tissue-specific calibration. Volumetric OA temperature mapping has demonstrated potential for utilization in photothermal therapy [24]. However, there are some drawbacks associated with temperature measurement using OA during laser surgery. These include poor axial and lateral resolution, a low acquisition rate for 3D measurements, and dependence on the repetition rate of the ablative laser. It should be noted that the accuracy of these methods has only been investigated below the coagulation temperature of the biological tissue. For temperatures exceeding 50 °C, there is non-linearity in the Grüneisen parameter and changes in the optical properties (absorption and scattering coefficients) of the tissue [25]. Consequently, the ability of OA techniques to accurately predict the temperature is limited to temperatures below the coagulation threshold. Optical coherence tomography (OCT) is another method with proven potential to measure temperature changes of tissue based on its photothermal expansion. OCT is a non-invasive, high-resolution, non-contact, and high-speed interferometric imaging technique capable of producing three-dimensional images of biological tissue based on the particular optical scattering properties of the various tissues [26, 27]. Recent studies have extensively investigated the advantages of using OCT integrated with laser ablation as a visual feedback system [28–30]. Phase-sensitive OCT (PhS-OCT) is an extension of conventional OCT that can detect axial deformation beyond its axial resolution, with high sensitivity and temporal resolution [12, 31, 32]. The high sensitivity of PhS-OCT makes it possible to detect the induced thermal expansion of the tissue caused by the absorption of the laser’s pulse energy during laser surgery. Therefore, the dependency of the optical path difference (OPD) on temperature can be used for photothermal tissue imaging during thermal therapy. Recent studies have proven PhS-OCT’s capacity to predict the temperature of a given sample for low-temperature changes (below the coagulation threshold) [33–36].

Although OCT’s potential to measure tissue temperature rise in the laser coagulation regime has been well-investigated, further investigation is required to predict the tissue’s temperature rise in the more complicated laser ablation regime. In this work, the principle of temperature rise of bone tissue during laser ablation using an Er:YAG laser is investigated. Followed by the proposed thermal model, a calibrated phase-sensitive OCT system that could potentially trigger the irrigation system by detecting the highest temperature in the area surrounding the laser-induced cut is introduced.

## Methods

**Fig. 1:**
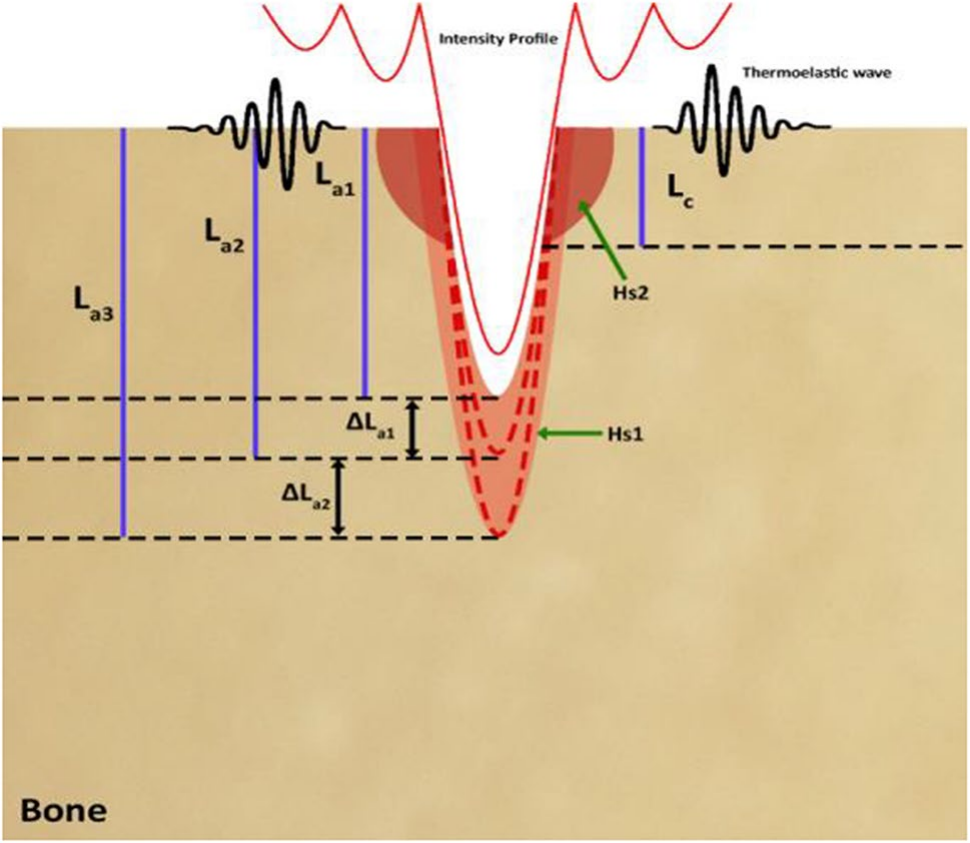
Schematic of the temperature rise of bone during laser ablation. Hs1 indicates the infinite number of heat disk sources, Hs2 represents the heat source on the surface of the bone. *L*_a_, Δ*L*_a_, and *L*_c_ are ablation depth, effective heat depth after ablation pulse, and effective heat depth on the surface of the bone, respectively

Figure 1 shows a schematic of the principles for a temperature rise of bone during laser ablation. The Gaussian distribution of the Er:YAG laser’s energy results in a cone-shaped ablation of the bone (Fig. 1). The geometry of the laser-induced cut denoted that the ablation volumes within the cut were not constant. Consequently, in a sequence of laser pulses, the difference between the ablated areas could be considered as an infinite number of disk heat sources, which is denoted by Hs1 in Fig. 1. This effect is similar to the friction between the drill bit and the wall of the cut, in traditional bone surgery [37]. The radius of the disk-shaped heat sources is equivalent to the laser-induced crater. In addition to the heat distribution from the borders of the cut, Er:YAG lasers often suffer from a low beam quality factor (*M*^2^), leading to the heating of an extended area on the bone’s surface. It measures the spatial distortion level of a laser beam in comparison to an ideal Gaussian beam of the same wavelength, with a value closer to 1 indicating a higher resemblance to the ideal Gaussian beam. In this study, the beam quality factor is measured as *M*^2^ = 22, which results in heating an extended area on the bone, beyond the borders of the laser-induced cut (Hs2 in Fig. 1) [38]. Hs2 indicates the heat source on the bone’s surface. The effective heat-diffusion length during a single laser pulse, Δ*L*_a_, was defined as $\Delta {L}_{\textrm{a}}\sqrt{4{Dt}_{\textrm{p}}}$ , in which *t*_p_ is the laser pulse-width, and *D* stands for the diffusion constant of the tissue [39]. Thermoelastic waves induced by absorbed laser energy, ablation depth (*L*_a_), effective heat source in between laser pulses (Δ*L*_a_), and effective heat depth induced by sidelobes of laser (*L*_c_) are also shown in Fig. 1.

### Induced temperature rise

The mechanism of hard tissue ablation using an Er:YAG laser can be considered a “photo-mechanical” procedure [40]. For bovine cortical bone, the effective heat-diffusion length (pulse duration of 300 μs) and laser penetration depth using an Er:YAG laser (*λ*=2.94 μm) are 18.6 μm and 4 μm, respectively [38, 41]. The beam size of the Er:YAG laser measured as 2.5 mm at the focal plane. Considering the cutting width of 1.1 mm on the surface of the bone, the heat source on either side of the crater was around 0.85 mm. Therefore, for the Hs2, due to the larger beam size value compared to penetration depth, the axial distribution of the heat ( *T*_axial_) is more effective and the temperature profile followed an exponential-like decay in the axial direction (Beer-Lambert law). However, for Hs1, which indicates the infinite number of heating disks, the spot size was assumed to be comparable to or less than the laser penetration depth, which meant that radial heat distribution (*T*_radial_) is dominant and followed a radial distribution of temperature. It is worth mentioning that in the proposed thermal model, it was assumed that the energy of the laser pulse is mainly consumed for tissue removal. Therefore, the temperature at any point in the tissue during laser osteotomy could be considered as follows:

1 $$T={T}_{\textrm{axial}}+{T}_{\textrm{radial}}$$

To determine the temperature rise (axial and radial) induced by these heat sources over time (∆*T*(*r*, *t*)), an analytical solution of the heat diffusion equation was used as follows [42]:

2 $$\frac{\partial\ \Delta \textrm{T}\left(\textrm{r},\textrm{t}\right)}{\partial t}-\alpha\ {\nabla}^2\Delta T\left(r,t\right)=\frac{Q_{\textrm{res}}}{\rho_0{c}_p}$$

In equation (2), α is the thermal diffusivity, *ρ*_0_ density and *c*_*p*_stands for the heat capacity of the bone tissue. To define the temperature rise of the tissue using equation (2), some parameters need to be considered. The residual heat that causes tissue temperature rise ( *Q*_res_) is difficult to measure inside a laser-induced cut. Furthermore, specific parameters should be known in advance. For instance, the water content in a human femur cortical bone varies between 21 and 17 % for humans aged 5 and 95 years old, respectively [43]. Furthermore, the micro-structural attributes of bone, including the size of pockets or pores within its structure, play an important role in affecting ablation efficiency. This influence arises from the changes in bone tissue’s thermal conductivity. The presence of water within bone can be found associated with mineral phase or free water (bulk water) [44, 45]. The pores within the calcified matrix are filled with bulk water, creating a network of interconnected channels. Consequently, these pores could serve as escape routes for laser-generated vapor, reducing pressure buildup that has the potential to diminish ablation efficiency [46]. Due to the aforementioned shortcomings, alternative solutions were sought for a tissue-specific calibration method.

### Phase-sensitive OCT

PhS-OCT is a functional OCT-based technique that can detect the axial deformation of a sample with a higher degree of accuracy than its axial resolution. PhS-OCT can provide additional contrast to conventional OCT images in terms of tissue changes over time. For this purpose, a highly phase-stable OCT system is required to isolate the changes in the phase of the tissue under investigation. In general, although spectral-domain OCT (SD-OCT) demonstrates higher inherent phase stability, faster imaging speed, and lower effect of fringes washout make SS-OCT more interesting for PhS-OCT applications [47]. Nevertheless, the mechanical principle of sweeping the wavelength in conventional swept lasers, such as polygonal scanning mirrors, and microcavity tunable lasers with microelectromechanical systems (MEMS), causes lower phase stability compared to the SD-OCT [48]. It is worth mentioning that several studies reported software and hardware-based solutions to improve the phase-stability of the measurements [49]. In this study, an akinetic swept source is utilized which provides a high-phase stability by eliminating the mechanical part, providing high-phase stability without the need for further post-processing.

During laser osteotomy, the tissue absorbs high-power laser energy and results in generation of heat. Consequently, the tissue expands and the optical path difference (OPD) changes on the surface of the tissue. At first, only the changes in OPD induced by Hs2, the heat source on the surface of the bone, are considered. The measured phase changes correspond to the integral of the refractive index within the depth of the tissue.

3 $$OPD\left({T}_0\right)={\int}_0^{L_0}n(T) dz=n\left({T}_0\right){L}_0$$

where *n*(*T*_0_) is the sample refractive index at the initial temperature, *T*_0_, and *L*_0_is the physical length of the sample. Assuming that the refractive index and the thermal expansion coefficient of bone tissue change linearly over time [50]:

4 $$n(T)=n\left({T}_0\right)+\frac{d n}{d T}\ \Delta T(z),\beta (T)=\beta \left({T}_0\right)+\frac{d\beta}{d T}\ \Delta \textrm{T}(z)$$

In addition, internal acoustic stress induced by the ablative laser causes thermoelastic deformation, which can be written as:

5 $$\rho \frac{\partial^2u}{\partial {t}^2}-\frac{E}{2\left(1+\nu \right)}{\nabla}^2u-\frac{E}{2\left(1+\nu \right)\left(1-2\nu \right)}\nabla \left(\nabla .u\right)=\frac{- E\beta}{3\left(1-2\nu \right)}\nabla \left(\Delta T\right)$$

In equation (5), *ρ* is the density of the tissue, *u* is the displacement, *E* is Young’s modulus, *v* stands for the Possion ratio, *β* is the thermal expansion coefficient, ∆*T* is temperature changes, and ∇ defines the displacement in the volume [51]. At the end of the acoustic stress, a quasi-steady-state equilibrium settles in. Therefore, the first term in equation (5) can be considered as zero. Then, the thermoelastic displacement can be described as:

6 $${u}_z=\frac{\beta \left(1+\nu \right)}{3\left(1-\nu \right)}{\int}_0^L\Delta Tdz$$

Therefore, the total tissue displacement can be derived from the summation of the thermo-optic and thermo-elastic terms. J. Kim et al., reported that in this thermal model, considering the axial temperature profile of the tissue heated by laser (Beer-Lambert law, Δ*T*(*z*) = ∆(*T*_0_) exp(−*μ*_*a*_*z*), axial displacement *δ*(*T*) can be written as [35]:

7 $$\delta (T)={n}_{T_0}\times L\left({T}_0\right)+\left[{n}_{T_0}\frac{1+v}{3\left(1-v\right)}\beta \left({T}_0\right)+\frac{d n}{d T}\right]\times \frac{\exp \left(-{\mu}_aL\left({T}_0\right)\right)}{-{\mu}_a}\ {T}_0+\left[{n}_{T_0}\frac{d\beta}{d T}+\frac{d n}{d T}\beta \left({T}_0\right)\right]\times \frac{1+\textrm{v}}{3\left(1-v\right)}\frac{\exp \left(-{\mu}_aL\left({T}_0\right)\right)}{-2{\mu}_a}\ {T}_0^2+\left[\frac{d n}{d T}\ \frac{1+\textrm{v}}{3\left(1-v\right)}\ \frac{d\beta}{d T}\ \frac{\exp \left(-3{\mu}_aL\left({T}_0\right)\right)}{-3{\mu}_a}\right]\varDelta {T}_0^3={c}_{a0}+{c}_{a1}\varDelta {T}_0+{c}_{a2}\varDelta {T}_0^2+{c}_{a3}\varDelta {T}_0^3$$

In equation (7), *C*_a0_, *C*_a1_, *C*_a2_, and *C*_a3_ are constant terms to correlate the optical path difference to the corresponding temperature rise induced by the ablative laser. Changes in the phase (*Δφ*) in PhS-OCT can be converted as $\delta =\frac{\lambda_0{\varDelta}_{\varphi }}{4\pi n}$.

For Hs2, where the conic-shaped laser-induced cut showed an infinite number of disk-heat sources, equation (4) for a specific depth could be written in a cylindrical coordinate. This indicated that the axial temperature profile (equation (7)) was modified by Hs2, which is a depth-dependent parameter; thus, the contribution of Hs2 adds more complexity to describing the bone’s axial temperature profile during laser ablation. Therefore, in this study, a calibrated PhS-OCT method capable of predicting the axial temperature profile of bone tissue induced by Hs1 and Hs2 is introduced. Equation (8) shows a simplified contribution of Hs1 and Hs2, which leads to the heat-induced displacement (*δ*^′^(*T*)) of tissue:

8 $${\delta}^{\prime }(T)=\left[{C}_{a0}+{C}_{Q0}\right]+\left[{C}_{a1}+{C}_{Q1}\right]\Delta {T}_0+\left[{C}_{a2}+{C}_{Q2}\right]\Delta {T}_0^2+\left[{C}_{a3}+{C}_{Q3}\right]\Delta {T}_0^3$$

### Experimental setup and signal processing

**Fig. 2:**
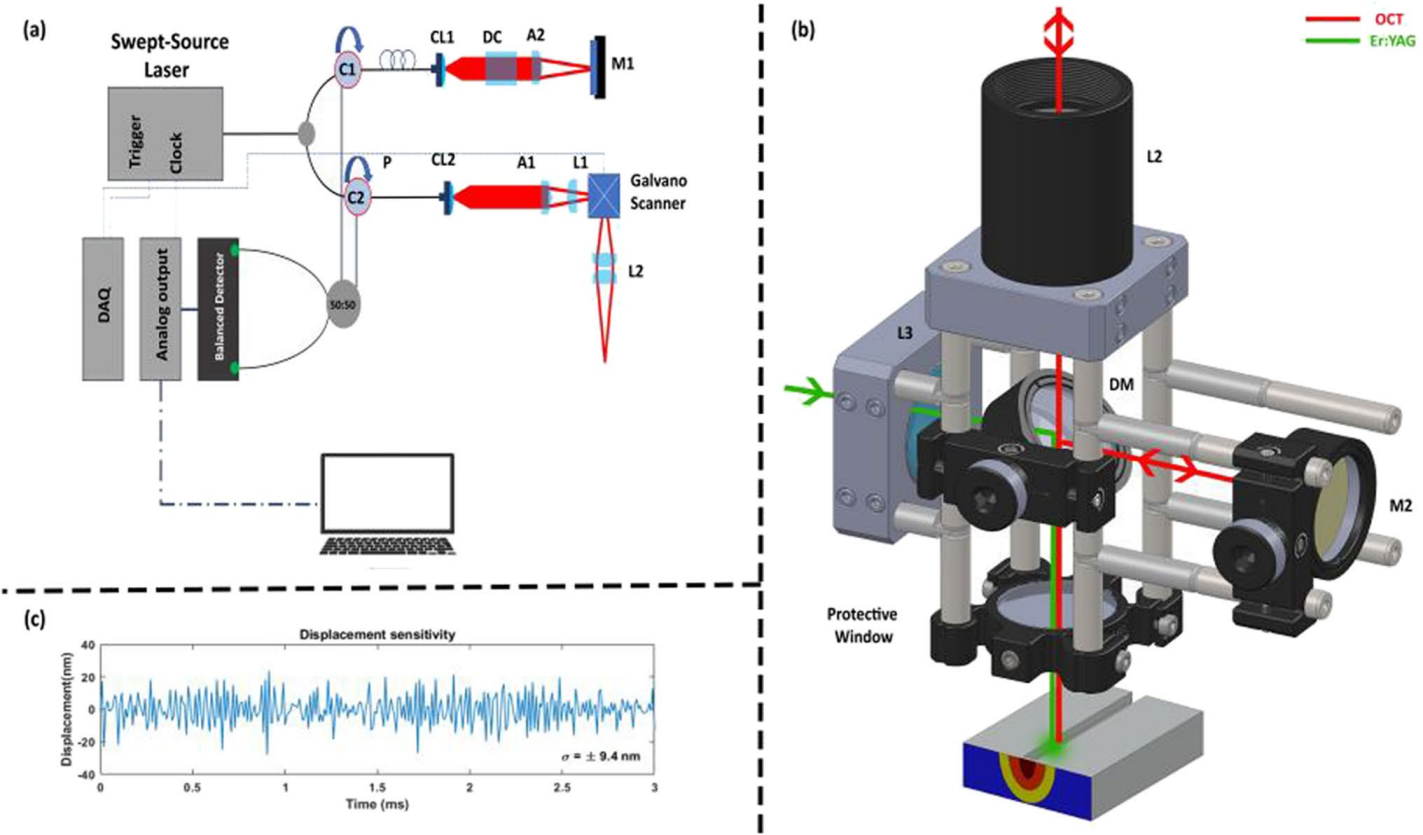
Schematic of the optical setup of the PhS-OCT system. **a** Schematic of the BLB-OCT system. **b** Integrated phase-sensitive OCT system and the ablative laser for PhS-OCT. P (polarization controller), CL (collimator), M1 and M2 (mirrors), A1 and A2 (axicon lens, apex angle of two degrees), L1 (achromatic lens, *f*=75 mm), L2 (achromatic pair lens, *f*=100 mm), and DM (custom-made dichromatic mirror)

A simple configuration of the tissue-specific PhS-OCT system is presented in Fig. 2, where Fig. 2a shows the Bessel-like beam OCT (BLB-OCT) system and Fig. 2b illustrates the modified sample arm for the PhS-OCT system. The configuration of the BLB-OCT has been described in [29]. The swept-source laser utilized in this experiment was a programmable akinetic swept-source laser (Insight Photonic Solutions, Inc.) with a central wavelength of *λ*_0_ = 1288 nm, a bandwidth of 60.9 nm, sweep rate of 104.17 kHz, and sample clock of 400 MHz (50% duty cycle). OCT’s wavelength is selected based on the density and highly scattering characteristics of bone and its components [44, 45]. This wavelength range exhibits less scattering and absorption compared to shorter wavelengths. The conjunction of reduced scattering and absorption, combined with the deeper penetration depth into bone tissue facilitated by BLB’s extended depth of focus, guarantees improved image contrast.

Imaging parameters were selected to provide sufficient speed, image quality, and imaging range, which are crucial features for a visual feedback system in laser osteotomy. The arrangement of the BLB-OCT system, shown in Fig. 2a, included two circulators (C1, C2) that directed the light to the sample arm and to the reference arm while also delivering the light collected from both arms to the balanced photodetectors (PDB480C-AC Thorlabs). In the reference arm, the polarization controller, collimator lens (F260APC-C, Thorlabs), dispersion compensation, axicon lens (AX-122C, apex angle of two degrees, Thorlabs), and mirror are represented by P, CL1, DC, AL, and M1, respectively. In the sample arm, the collimator lens, axicon lens (same as the reference arm), achromatic doublet lens (*f*=75 mm, AC127-075-C, Thorlabs), and achromatic pair lens (*f* = 100 mm, Edmund optics, #47-302) are sequentially indicated by CL2, A2, L1, and L2. In Fig. 2b, the Er:YAG laser combined with the OCT laser using a custom-made dichromatic mirror (DM, 97% reflection at 2.94 μm, and transmission of > 90% at 1300±75 nm), and focused on the sample using a *f* =75 mm calcium fluoride lens (L3, L5042, Thorlabs). The standard deviation of the phase sensitivity measured using a microscope glass slide (1 mm) is measured as ≈ ± 9.16 radians over 3 ms. Figure 2c illustrates the corresponding measured displacement sensitivity with a standard deviation of ± 9.4 nm.

To calibrate the tissue-specific PhS-OCT, phase information (using PhS-OCT) and the value of the temperature rise should be recorded simultaneously. In this experiment, a thermal camera is used as a reference. Despite the difference in the pixel sizes of these systems, to the best of our knowledge, an infrared camera is a standard technology that can remotely measure the temperature rise in the ablation regime (*T*>50 °C). The region of interest in selected based on the reference point (ablation crater) and correlation of the pixel sizes in both systems (170-μm thermal camera, 18 μm OCT). Then, the raw data from the PhS-OCT system and the thermal images from the thermal camera were recorded continuously, starting just before laser ablation, and ending when the sample returned to room temperature. The pre-processing steps for the PhS-OCT included background subtraction, spectral shaping, and fast Fourier transformation. Then, to evaluate the phase changes, phase information of the sequential A-scans between the frames (1000 A-scans) was extracted and converted to the axial displacement. To remove the bulk phase fluctuation, cumulative phase information on the sample was subtracted from the reference value acquired by the calibration arm in Fig. 2b. To compare the temperature, rise and phase changes within the lasing window, a re-sampling was performed so that both systems would have the same sampling rate. Finally, by fitting a polynomial of degree three to the cumulative phase and temperature, the constants mentioned in equation (8) are determined. Having calculated a constant parameter with which to correlate temperature rise and cumulative phase change, the temperature rise corresponding to the phase change using the fzero function in Matlab software (version R2021b) is estimated.

### Tissue-specific phase calibration

Equation (8) denotes the correlation between the temperature rise and changes in the optical path length. Once the fitted coefficient has been established, the temperature rise of the bone at the same depth as the laser-induced during the calibration step can be calculated. Consequently, the calibration coefficient could be used in place of the thermal camera to predict the temperature rise of the bone. The unique advantage of the proposed tissue-specific calibration method is that allows temperature rise of a sample without any initial information about the tissue.

### Sample preparation

For this study, bovine femur slices were purchased from a local supermarket. The soft tissue was removed, and the bone was frozen for one day. Then, the edges of each bone were flattened using a bone saw. Ablating bone on the flattened edge made it possible to monitor the temperature inside the bone tissue. After cutting, each bone was washed carefully with tap water to remove surface debris.

## Results

**Fig. 3:**
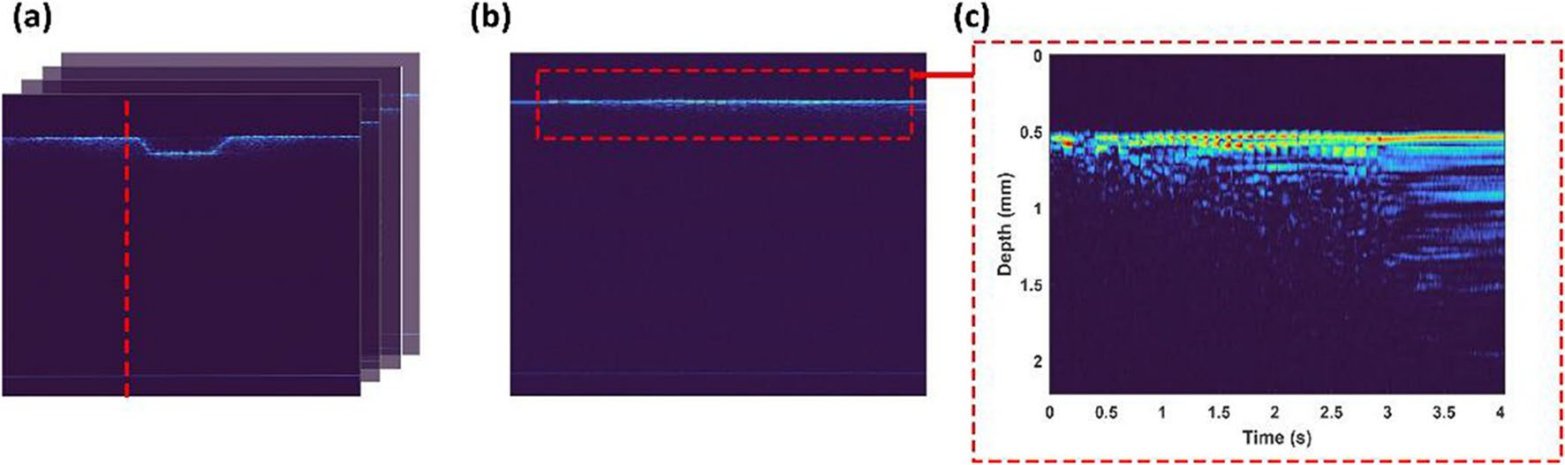
Induced photothermal expansion of the bone during laser osteotomy. **a** intensity-based image, **b** M-scan image corresponds to the A-scan illustrated by dashed red-line in **a** over time, and **c** magnified photo of the induced displacement of the bone tissue from **b**

Figure 3 illustrates the photothermal expansion of the bone during laser osteotomy using an Er:YAG laser with a repetition rate of 10 Hz and an energy per pulse of 230 mJ. Figure 3a shows the intensity-based images of the recorded laser-induced cuts. The line at the bottom of the image represents the reference line, which was used to remove phase fluctuation. Figure 3b illustrates the extracted A-scan (red dashed line in Fig. 3a) from a sequence of B-scans over time (M-scan), and Fig. 3c corresponds to a zoomed-in version of Fig. 3b, where induced displacement by absorption of the laser pulse energy is visible.

**Fig. 4:**
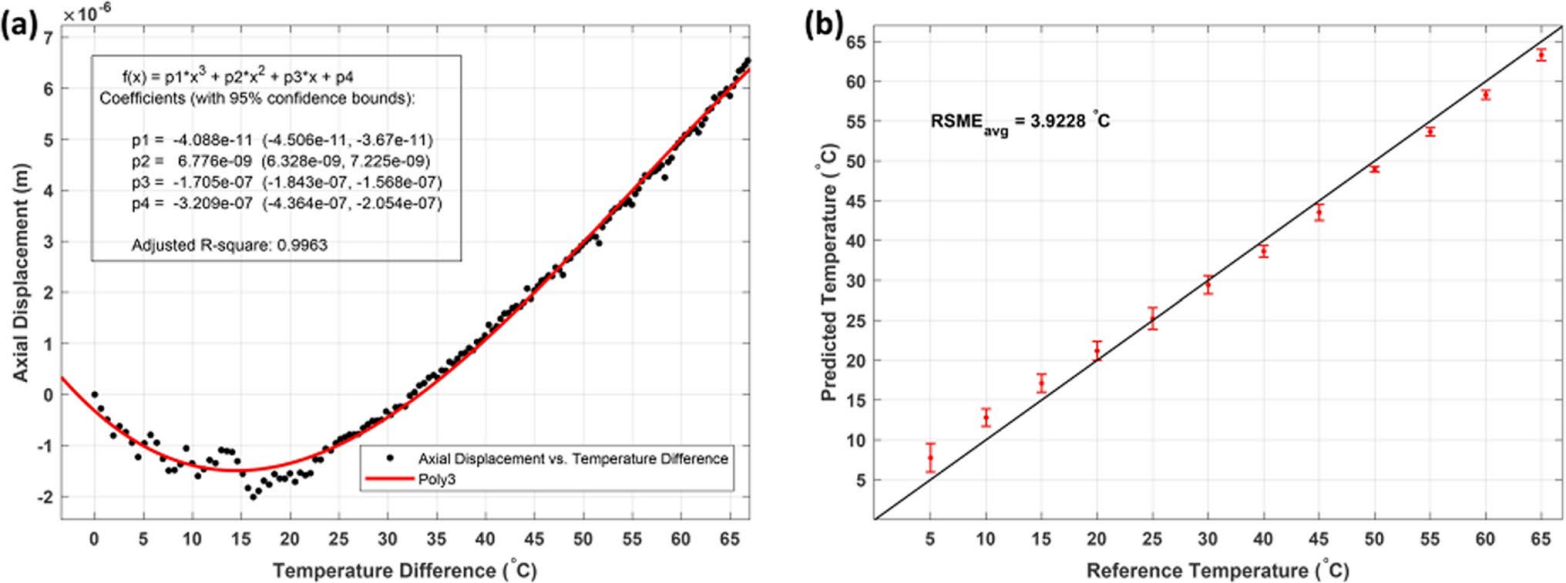
**a** Polynomial of degree three defines the calibration coefficient for correlating the temperature rise and corresponding axial displacement. **b** The errors of the predicted temperature are shown using the coefficients acquired during the calibration process and used for three different spatial points on the same bone samples

As mentioned above, during the calibration process, simultaneous recording of the bone’s phase change and temperature change during laser osteotomy is required. Figure 4a demonstrates the calibration process performed on the bone, where the *x*-axis is the recorded temperature difference, and the *y*-axis shows the axial displacement.

In Figure 4a, the red line shows the polynomial degree three, which correlates the cumulative axial displacement and temperature differences. Following the calibration process and having acquired the required coefficient, equation (8) was used to predict the tissue temperature rise. Figure 4b shows the predicted temperature rise for three different spots on the bone tissue and the differences compared to the temperatures recorded by the thermal camera. The comparison was performed at the same distance to the crater’s center as for the calibration experiment.

**Fig. 5:**
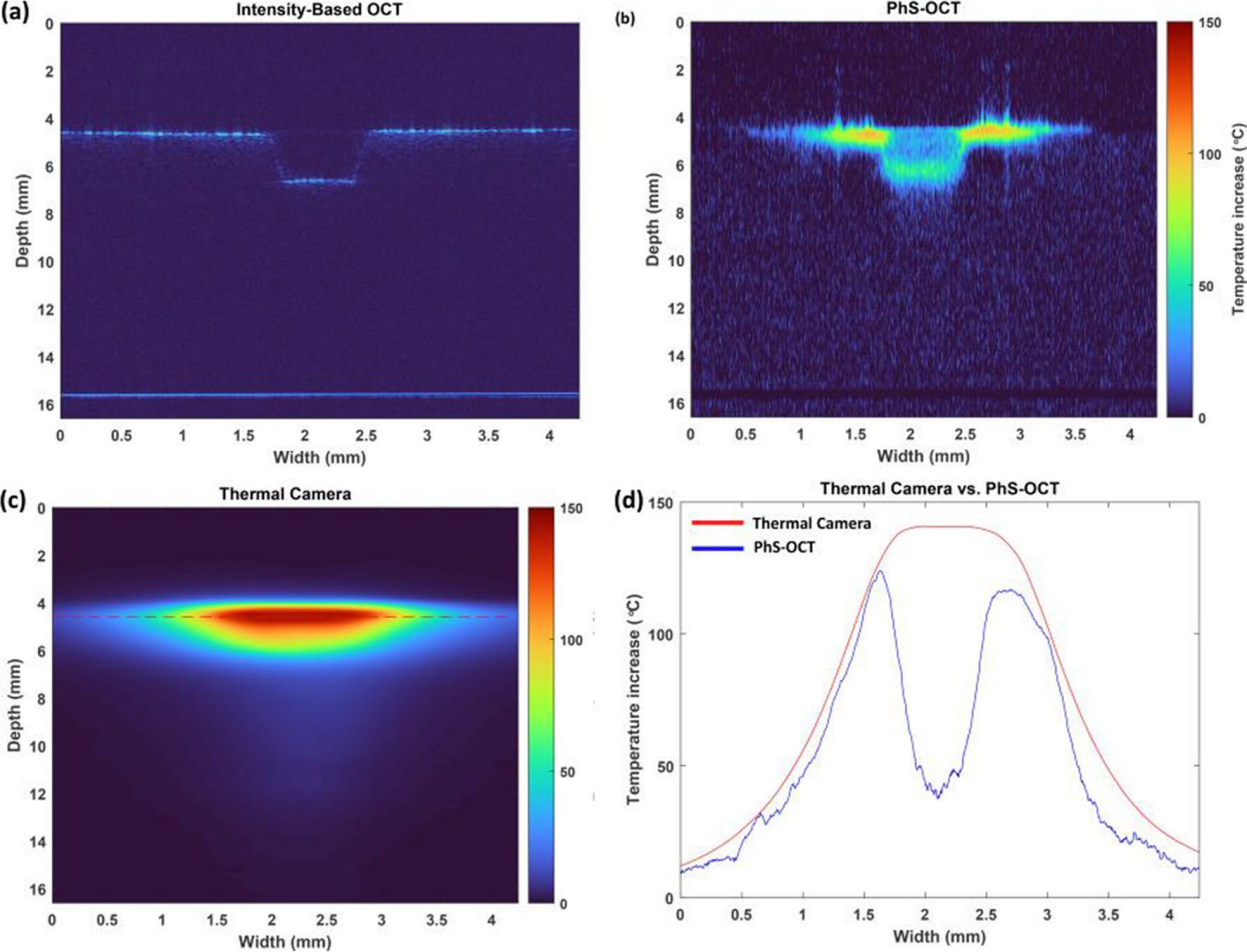
Monitoring the depth and temperature rise of the bone during laser osteotomy using an Er:YAG laser (10 Hz, 230 mJ energy per pulse). **a** Intensity-based image of the crater, **b** PhS-OCT image of the cumulative temperature rise, **c** reference temperature using a thermal camera, and **d** comparison of the predicted temperature and temperature rise on the surface of the bone. The duration of the experiment was 3.7 s

In addition to the single-point temperature measurements, the potential of using the coefficients acquired during the calibration process to predict tissue temperature rise in the B-scan OCT images (Fig. 5) is investigated. To investigate the two-dimensional temperature map, a thermal camera continuously monitored the bone’s temperature. Figure 5a corresponds to the intensity-based OCT image, which shows the depth and shape of the laser-induced cut in real time. Figures 5b and c demonstrate the predicted temperature map using PhS-OCT, and the reference temperature map using the thermal camera, respectively. Figure 5d compares the predicted temperatures and reference temperatures on the surface of the bone.

## Discussion

Although laser osteotomy offers several advantages over conventional methods for bone surgery, induced thermal damage is a major challenge that limits its application in clinical settings. In this study, the potential of a PhS-OCT system to provide feedback for controlling irrigation and real-time feedback for monitoring the depth of the laser-induced crater (provided by a conventional OCT system) is demonstrated. Figure 3 illustrates the photothermal expansion of the bone during laser osteotomy, which underlies the proposed PhS-OCT method for determining tissue temperature rise. Here, in addition to tissue expansion, changes in the attenuation coefficients of the bone due to temperature increase are also visible. The latter of which can also be used to determine the temperature rise of the tissue [52]. The PhS-OCT calibration step is characterized by simultaneously monitoring the temperature rise of the tissue (using a thermal camera) and its photothermal expansion (using an OCT system) and defining the correlation between them using equation (8). Figure 4a shows the process of extracting the coefficient by fitting the recorded temperature difference and axial displacement of the bone using equation (8). An adjusted *R*-square of 0.9963 using a polynomial of degree three demonstrates that the fitted parameter can predict the temperature rise of the tissue. An initial decrease in the cumulative phase was followed by an increase in the bone’s temperature, due to the decrease in the refractive index of the water and the expansion of the bone’s structure demonstrated in Fig. 4a [53]. During the experiments, it was noticed that this decreasing tendency varied among different bone samples. Fresh bone, for example, had the greatest decrease in the phase, while dried bone did not show any initial decrease but followed an increasing trend from the first laser pulse. Extracting the attenuation map of the tissue from OCT images could potentially prevent this issue by monitoring and classifying the status of bone samples (hydrated, dehydrated, and carbonized). The preliminary results demonstrated the potential of utilizing attenuation map to classify the status of bone, however, identifying the reasons for variation in attenuation profile can be challenging since it could arise from the refractive index change of the tissue or the reduced water content due to high temperatures. It is worth noting that dependency of the refractive index on temperature limits this comparison in the ablation regime. This requires further investigation to calculate the refractive index of bone as a function of temperature.

The acquired calibration coefficients were used to predict the temperature rise of three different laser-induced cuts at the same distance to the center of the crater as the calibration experiment (Fig. 4b). The average value of root-mean-square error for the three experiments was estimated as 3.9 °C. The main error has been observed at the beginning of the laser ablation process, which attenuation map can assist in reducing this error by classification of the state of the bone. Moreover, the generation and accumulation of debris around the incision are considered contributing parameters affecting the precision of temperature prediction. The interaction between debris and subsequent laser pulses introduces variations in phase comparison on the bone’s surface. Utilization of high-pressure air can potentially reduce this error. Additionally, the proposed calibration approach currently focuses on evaluating the temperature of a single pixel, However, extending this calibration to encompass all the pixels on the surface could not only enhance accuracy but also enable the determination of the relationship between tissue-specific parameters and the temperature profile (spans from the crater’s edge to more distant spatial point). The primary objective is to utilize it as a feedback system for the irrigation process during laser osteotomy. This can be achieved by defining a desired threshold and considering the error.

Finally, further investigation was conducted to determine the two-dimensional temperature rise of bone using the calibration coefficients acquired at a single point (Fig. 5). A cross-sectional intensity-based OCT image of the laser-induced crater is presented in Fig. 5a, where both the depth and shape of the cut are visible. The calculated temperature rise of the bone in this experiment is given in Fig. 5b, and the reference temperature recorded by the thermal camera for comparison can be seen in Fig. 5c. The image acquired by the reference indicates that the highest temperature rise occurred inside the laser-induced cut, where the tissue had been removed. In contrast, due to the working principle of the PhS-OCT, the highest temperature rise is detected at the borders of the crater. This unique feature, the ability to detect temperature where tissue exists, could potentially enhance ablation efficiency compared to utilizing a thermal camera for controlling an irrigation system [54]. The structure of the cut is not visible in the infrared camera image, making it challenging to find a suitable reference point on the surface. However, the PhS-OCT system can visualize the cumulative temperature rise of the bone during laser-induced ablation and preserve the crater’s shape. The phase noise and limited image range of OCT inside the tissue cause incorrect temperature detection. These errors can be minimized by selecting the region of interest in the PhS-OCT images based on the level of the signal in the intensity-based images of the OCT. Moreover, this process can also improve the calculation time required to produce PhS-OCT images. Figure 5d compares the temperature profiles on the bone’s surface, which implies the difference between the detected temperature rise inside the cut in PhS-OCT method and thermal camera. Since the principle of the PhS-OCT is to follow the cumulative temperature changes over time, the removal of tissue inside the cut results in the PhS-OCT showing the last temperature reading at that point when the tissue was present.

The proposed method has the potential for use as a feedback mechanism by which to trigger pulsed irrigation during laser surgery (instead of continuous irrigation). Furthermore, calibrated PhS-OCT has the unique advantage of predicting the temperature rise of the tissue during laser ablation without prior knowledge of the opto-mechanical and thermo-optic properties of the target tissue, which are usually difficult to obtain. This method could be used in endoscopic applications and minimally invasive surgery, where a thermal camera would not be suitable. Although the PhS-OCT shows several unique advantages, it also has some limitations. To ensure the same configuration of the temperature distribution for Hs1 and Hs2, the distance between the bone and the focusing lens of the Er:YAG laser should be kept constant. Likewise, since this method uses induced cumulative phase changes, a proper unwrapping method is required to maintain the accuracy of this method. When transitioning from ex vivo to in vivo experiments, it is crucial to consider various parameters, including the presence of blood during surgery, the existence of other instruments in the surgical room, and the patient’s movements. These factors have the potential to introduce noise into the phase measurements. To address this, the implementation of a real-time tracking and calibration algorithm is proposed. For instance, repositioning and real-time calibration can be performed by implementation of an external point temperature measurement (such as a miniaturized infrared camera). This continuous monitoring and adjustment of the acquired calibration parameters could potentially serve to enhance precision and maintain accuracy. In addition, the suggested improvement ensures the safety of laser surgery which is desired for medical applications.

## Conclusion

Lasers have brought many advantages to the medical field of osteotomy; however, they are not without drawbacks. During laser osteotomy using Er:YAG lasers, thermal damage is usually prevented by applying a continuous irrigation system, which, consequently, may lead to water accumulation reducing the ablation efficiency. The experimental results demonstrate the potential for utilizing the calibrated phase-sensitive OCT system as a temperature feedback mechanism, with an average root-mean-square error of 3.9°C. The introduced method is based on acquiring the tissue-specific parameters during calibration step by correlation of the measured temperature (acquired by thermal camera) and photo-thermal expansion (using phase-sensitive OCT). This feedback, combined with real-time visualization of the depth of cut using intensity-based OCT images (as shown in Fig. 5), offers a comprehensive solution to mitigate thermal damage and monitor the depth of laser-induced cut during laser osteotomy procedures.

Improving the accuracy of temperature prediction by integration of an online calibration algorithm is among our main interests for the future. Furthermore, despite the fact that OCT can provide three-dimensional images of the tissue, limited comparison means preventing further investigation of the temperature map which needs to be addressed. Independence of the phase-calibration method offers additional benefits such as acquiring the linear thermal expansion coefficient/refractive index of the tissue as a function of temperature.

## Data Availability

Data underlying the results presented in this paper are not publicly available at this time but may be obtained from the authors upon request.
